# The effect of reading strategy use on online reading comprehension

**DOI:** 10.1016/j.heliyon.2024.e24281

**Published:** 2024-01-12

**Authors:** Anita Habók, Tun Zaw Oo, Andrea Magyar

**Affiliations:** a Institute of Education, University of Szeged, MTA–SZTE Digital Learning Technologies Research Group, Szeged, Hungary; b Doctoral School of Education, University of Szeged, MTA–SZTE Digital Learning Technologies Research Group, Szeged, Hungary; c Hungarian University of Agriculture and Life Sciences, Institute of Education, MTA-MATE Early Childhood Research Group, Kaposvár, Hungary; d MTA–SZTE Digital Learning Technologies Research Group, Center for Research on Learning and Instruction, University of Szeged, Szeged, Hungary

**Keywords:** Online reading, Reading strategy, Reading comprehension, Language arts, Language attitudes

## Abstract

In the current age, digital technology is rapidly changing daily routines, and young people today spend most of their time using various digital tools. Therefore, traditional reading of the printed page is being transformed into digital reading of online texts among students. Thus, online reading strategies have become crucial for their development in online reading performance. This study aimed to investigate the effects of online reading strategies used by lower secondary students on reading comprehension achievement. It conducted an online survey of reading strategies, involving three types of reading strategies, global, problem-solving, and support. The study recruited 4527 students at the lower secondary school level in Hungary. The study examined the students' attitudes toward literature and grammar in their native language (L_1_), use of online reading strategies, reading comprehension skills, and language arts achievement as well as examining the relations between them with various methods of analysis (descriptive/inferential, Rasch and path analyses). The findings demonstrated that the students' problem-solving strategies (from among the three reading strategies) exerted significant and positive impacts on reading comprehension. Additionally, the students’ attitudes toward L_1_ had a positively significant effect on their use of online reading strategies and language arts achievement and an indirect effect on reading comprehension skills. The study also found a significant relationship between language arts achievement and reading comprehension achievement. Therefore, this study is beneficial for language teachers in helping students improve reading comprehension skills.

## 1 Introduction

Reading is a lifelong learning skill for students in and out of school and throughout their lives. Furthermore, reading can bring opportunities for school success and job opportunities [1]. Despite its importance, students struggle to use effective reading strategies for a holistic understanding of reading texts. In this regard, reading strategies provide students with skills to comprehend reading passages; thus, such strategies are crucial to the students’ language learning process [[2], [3], [4], [5]].

In the modern age, digital and online tools have become important resources for language acquisition and educational achievement [[6], [7], [8]]. Additionally, students spend the majority of their time outside school, navigating the Internet via mobile phones, laptops, computers, and other electronic devices; thus, the classroom, which does not encourage online learning, lacks relevance to the real-world situation [9]. The current nature of literacy has changed the learning process due to the emergence of new technological tools [10]. From the perspective of the change in literacy, the reading comprehension process is crucial for all language learners because new learning skills, reading strategies, and enthusiasm are required for a thorough textual reading [11]. Moreover [12], asserted that online reading has become a crucial source for readers in line with the importance of computers and the Internet for users worldwide.

Such rapid technological change is a reminder for traditional readers of the printed page that the normal decoding required to comprehend the linguistic features of a reading passage is insufficient. Consequently, it is crucial for learners to possess online reading skills and strategies to assist in grasping the meaning of online texts [11]. Reading online is different from traditional reading or reading the printed page because new reading strategies can promote an understanding of online reading texts [13]. Traditional reading refers to the act of reading print materials, such as books, newspapers and magazines, without the use of digital or electronic devices [14]. In contrast, reading online involves a blend of traditional reading and the use of information and communication technology (ICT), necessitating the adoption of various reading approaches, such as global comprehension, problem-solving and support reading strategies [15]. According to the Programme for International Student Assessment (PISA) survey for 2009–2018, traditional reading of print books, newspapers and other such material is lagging behind reading online through the world wide web and the Internet due to a massive increase in the use of technology among young learners worldwide [16,17].

Using online reading strategies in the classroom can increase students' active participation in the learning process [18]. These strategies have therefore become important for reading comprehension achievement [10]. They are recommended for use in reading online texts to help motivate interest in reading [19]. Several studies (e.g., Refs. [1,[20], [21], [22], [23], [24]]) investigated reading strategies used by students for print texts and even in learning English as a foreign language (EFL; e.g., Refs. [10,[25], [26], [27]]). Nevertheless, studies that have examined online reading strategies for learning in one's native language (L_1_) are few. This research gap encourages the present study to explore whether the use of online reading strategies among students in Hungary predicts language learning achievement in reading comprehension.

The significance of this study lies in its potential to inform language teachers and materials developers as they design effective instructional approaches for developing online reading skills. Investigating reading strategies and their impact on comprehension can contribute to the development of effective reading instruction. Understanding which strategies are most beneficial for online reading comprehension can help educators optimize learners’ reading experience and improve their reading skills. In the digital age, online reading has become increasingly prevalent [9]. Studying online reading comprehension in a specific language, such as Hungarian, can shed light on how individuals navigate and comprehend digital texts [28,29]. This knowledge can contribute to the development of digital literacy skills and help individuals engage with online content effectively. With its focus on the Hungarian language (L_1_), this study can provide insights into the unique challenges and strategies employed by Hungarian students when reading online texts at the lower secondary level. Understanding how specific language features, grammar or vocabulary impact online reading comprehension can inform the design of language-specific reading materials and instructional approaches [30].

Therefore, this study also plans to fill a crucial gap in the existing literature by examining three types of online reading strategies used by lower secondary students (global reading, problem-solving and support reading strategies) and investigating the relations between them as well as students' attitudes toward literature and grammar in their native language (L_1_), reading comprehension skills and language arts achievement. Studying students' attitudes toward literature and grammar in their L_1_ is crucial, as it reflects their overall language proficiency and intrinsic motivation toward language learning [31]. Additionally, exploring students’ use of online reading strategies allows us to better understand their adaptive learning behaviours in a digital reading environment [32]. By studying the relations between these factors, this study provides a comprehensive analysis of the interplay between online reading strategies, attitudes, language arts achievement and reading comprehension outcomes.

The present study examines the impact of reading strategy use specifically in the context of Hungarian (L_1_) reading comprehension on an online platform. While the use of reading strategies in English as a foreign/second language (L_2_) reading has received considerable attention in the literature (e.g. Refs. [22,26,27,[33], [34], [35], [36], [37], [38], [39], [40], [41], [42]]), there is a scarcity of research that examines reading strategy use in L_1_ online reading. This research gap highlights the need for an investigation into the reading strategies employed by L_1_ readers and their effect on online reading comprehension.

## 2 Literature review

### 2.1 Online reading literacy

Nowadays, students spend a great deal of time reading, searching for information and communicating on the Internet for various purposes (e.g., schoolwork and social interaction; [11]). In the digital age, online reading literacy encompasses the ability to seek, expand, apply and create new information online [[9,43]]. Furthermore, online reading literacy has become a crucial source of information for teachers and students [6,19]. Hence, online reading literacy is a prominent and appropriate skill for students in language learning [44].

For students, there are distinct differences between reading online texts and reading texts on paper. In online reading, students acquire information from webpages in different forms, such as hyperlinks, colourful texts, attractive images, interesting videos, related sounds and diverse animations [45,46]. Online reading literacy involves nonlinear reading hypertext, multimedia reading text and interactive reading texts not only to support reading comprehension but also to solve struggling problems during the reading process [27].

Online reading literacy includes five major functions, setting key questions, searching for information to answer these questions, criticising the resultant information, integrating meaningful information and conveying information [47]. In fact, online literacy can stimulate curiosity by supporting various topics with varied levels of reading difficulty, by scaffolding reading comprehension with background language and knowledge support through hyperlinks, and by offering online multimodality and ease of access to required information with a few clicks [9].

Furthermore, language learners become more interested in online reading than they do in offline reading because online texts are more interesting and stimulate curiosity and creative thinking [48]. [49] also contended that online literacy can promote student preference for two reasons: (1) students gain faster and easier access to required information through online materials compared to print materials and (2) webpage contents can provide an abundance of helpful and wide-ranging sources of information compared to offline materials.

Students need the skill of adjusting reading patterns and objectives in new online reading environments [27]. Online reading literacy is more enjoyable for students and can even arouse interest in learning because of its technology, which is mainly based on computers and the Internet. Moreover, online literacy can help students improve reading fluency due to online access to new tools, resources and communities [50,51].

In addition to the advantages for students of online reading noted previously, there are also some challenges that online readers face. As the shift from traditional paper-based printouts to modern digital media has occurred, acquiring and mastering the skill of online reading has become a great challenge for students [11]. Another difficulty is the need to make choices on which link to click on when confronted with multiple embedded hyperlinks while engaging with nonlinear hypertexts [36]. Furthermore, according to Refs. [10,52], it is evident that online readers require not only skills related to online reading but also strategies specifically designed to enhance their reading comprehension in digital contexts.

### 2.2 Reading strategies

Reading strategies pertain to the purposeful endeavour of interpreting the meaning of reading texts. Different reading strategies encourage various means through which students receive information [53]. Such strategies are a crucial tool for students and can provide them with fundamental benefits not only in improving comprehension but also in enjoying reading texts [54,55]. Among the recognized reading strategies that involve cognitive and metacognitive methods, cognitive reading strategies refer to actions taken to perceive meaningful information from the reading text and metacognitive reading strategies denote higher-order thought, such as planning, monitoring and evaluating, for the transformative learning of the reading text [35,36]. According to Ref. [56], metacognitive learning approaches should be encouraged because they aid in transformative learning among students.

As regards metacognitive reading strategies, the majority of outstanding students use relatively common reading strategies: (1) identifying and considering reading topics, (2) setting reading objectives, (3) reading the text thoroughly, (4) reflecting on their comprehension and (5) promoting an increased understanding [27]. Furthermore [11], proposed several reading strategies, such as (1) understanding the reading objective, (2) grasping key information from the reading passage, (3) relating links to the information involved, (4) reflecting on activities that have occurred to enhance comprehension, (5) self-evaluating comprehension, (6) modifying one's comprehension if necessary and (7) developing a complete understanding of the reading text. These reading strategies are also classified into three types of approaches, bottom-up, top-down and interaction between both approaches [57]. Diverse students also perform different types of reading (skimming, scanning, intensive reading and extensive reading) based on the objectives of the activity [58].

Furthermore, reading strategies can be used in online learning environments if students are provided with support for various learning opportunities during the reading comprehension process [59]. As online texts may include nonlinear hypertext and multimedia and interactive texts, students are generally dependent on their current reading strategies and transfer strategies for print texts to online texts [27].

[60] used a web-based program that focused on metacognitive approaches, such as global, problem-solving, supportive and socio-affective reading strategies. Furthermore [12], conducted a study on online reading strategies and investigated their use among students by dividing them into three types, the global, problem-solving and support reading strategies. Similarly [42,61], studied the use of reading strategies for online reading text. The results indicated the use of three major online reading approaches: the global, problem-solving and support reading strategies.

Other studies [22,23,26,62] focused on the use of reading strategies among students for comprehension in language learning. These studies used the online survey of reading strategies (OSORS) and highlighted three approaches, the global, problem-solving and support reading strategies. Based on these studies on the use of online reading strategies, the present study concludes that the common approaches are the global, problem-solving and support reading strategies.

### 2.3 The global reading strategy

This strategy denotes a reader's intended and planned approach to monitoring the entire process of reading comprehension. It is also one of the techniques that readers use to manage reading (e.g., readers begin with an objective in mind while reading texts or deciding on which aspects to focus on or ignore; [22]). It helps students in monitoring the reading process, reviewing reading content, examining its suitability to content, observing the characteristics of reading passages (e.g., coherence, length and organisation), and interpreting the meaning of the reading text [63].

When we evaluate reading comprehension, reading strategies play a crucial role as mediators between students' reading motivation and their ultimate achievement in reading comprehension, as emphasised by Ref. [41]. Moreover, these reading strategies can also serve as intermediaries between teachers' reading instruction and students' reading comprehension achievement [64]. Additionally [65], agreed that students' adoption of learning or reading strategies, along with their enjoyment of reading, can exert both direct and indirect influences on their performance in reading comprehension. Moreover [66], stressed the relationship between students' reading strategies, encompassing global comprehension, problem-solving and support techniques, and their language profile, which includes factors like language attitudes, proficiency in known languages and familiarity with other languages. This linkage between these reading strategies and students' achievement in language arts is noteworthy. In fact, one study by Ref. [38] suggested that these strategies represent an integral component of language arts achievement, with a notable influence on overall reading comprehension achievement. Moreover [40], recommended that teachers motivate their students to use digital tools when engaging in online reading, as this can lead to significant improvements in both language arts proficiency and reading comprehension. It is evident that students' abilities in reading and language arts are closely intertwined, and this interplay significantly impacts their achievement in reading comprehension, as also underscored by Ref. [33]. As such, it becomes imperative to investigate whether these reading strategies similarly act as mediators between students' attitudes toward their language skills and their overall achievement in reading comprehension. In addition, it is of paramount importance to acknowledge the significance of language arts achievement within the broader context of students’ reading comprehension process.

### 2.4 The problem-solving strategy

This strategy pertains to procedural actions based on challenges that emerge from a reading passage. It indicates that readers intend to solve upcoming problems in the reading process (e.g., if readers experience unfamiliar text formats, they use maps that outline the text structure and other related signals, such as titles, clues, colours and arrows; [13]). It also consists of making inferences from unfamiliar words, adapting to one's unique reading rate, organising confusing parts and refocusing on the reading passage to achieve understanding [[26,67]].

### 2.5 The support reading strategy

This strategy is the basic support mechanism of online reading programs, which aids in the reading comprehension process. It pertains to approaches for obtaining help or support from the reading text (e.g., readers attempt to translate reading passages into the target language or to paraphrase them into easily understandable ones; [68]). This strategy targets the expansion of comprehension; thus, students can use dictionaries, take notes and highlight key information [63].

### 2.6 Self-regulated learning and online reading strategies

Self-regulated learning (SRL) is an umbrella term with a significant number of variables that affect learning. On a practical level, individuals actively pursue knowledge by gathering information and making informed decisions about learning methods and strategies that they believe will enhance their understanding [69]. When examining the relation between SRL theory and online reading strategies (especially the global, problem-solving and support reading strategies), we can explore how SRL theory provides a framework for understanding the role of learners’ self-regulatory processes in using these strategies in online reading comprehension. What follows is a breakdown of how SRL theory relates to each of these strategies. Within the framework of SRL theory, students who employ a global reading strategy actively engage in setting goals and monitoring their understanding throughout the online reading process [70]. They may set an overall comprehension goal for the text and continuously assess their progress toward that goal. By doing so, learners regulate their cognitive processes and adjust their reading strategies accordingly to ensure they are comprehending the text at a global level. As for problem-solving strategies, in the context of SRL theory, learners who use problem-solving strategies demonstrate self-regulation by monitoring their comprehension and recognising when they encounter obstacles or gaps in their understanding [71]. They employ metacognitive processes to identify problem areas, apply appropriate cognitive strategies (e.g., inferencing and critical thinking) and self-correct their understanding to resolve the comprehension challenges they encounter in online reading. Furthermore, in the context of SRL theory, learners who use support reading strategies exhibit self-regulation by recognising their need for additional support, seeking out appropriate resources and applying these external aids effectively in online reading [72]. They demonstrate metacognitive awareness by identifying their comprehension gaps and using external support to scaffold their understanding.

Overall, SRL theory emphasises the active, goal-directed and self-regulated nature of learning. When it is applied to online reading strategies, learners who engage in self-regulatory processes are more likely to set specific goals, monitor their comprehension, select appropriate strategies (including the global, problem-solving and support strategies) and adapt their strategies based on their understanding and reading goals. By using these self-regulatory processes, learners enhance their metacognitive awareness, optimize their cognitive engagement and ultimately improve their online reading comprehension. It is important to note that while the relation between SRL theory and online reading strategies is plausible, empirical studies specifically focused on this link in the context of online reading comprehension would provide more concrete insights.

SRL theory covers various aspects of learning, including cognitive, metacognitive, motivational, emotional and behavioural components, making it a comprehensive framework that influences students' learning and academic achievement [73]. Therefore, in the context of online language learning, emotional factors or attitudinal factors play a significant role in students' inclination to use technology or to choose one or more online reading strategies. Thus, attitudinal factors are also important for considering students’ online reading strategy use and their reading comprehension and language learning achievement.

### 2.7 Assessment of online reading strategies

[61] developed a four-part survey that contains 35 items to assess the use of online reading strategies among 100 undergraduate students. The questionnaire presented three main categories of assessment using 36 items (18, 9 and 9 items for the global, problem-solving and support reading strategies, respectively). The study found that students most frequently used the support reading strategy [12]. conducted a study using the survey of reading strategies (SORS) to assess the use of reading strategies among 131 undergraduate EFL students in San Jose, Costa Rica. The SORS assesses three types of reading strategies: global (13 items), problem-solving (8 items) and support (9 items). The finding demonstrated that the students mainly availed themselves of the problem-solving strategy in online reading.

To assess online reading strategies [74], used the online reading strategy survey with 36 statements (16, 11 and 9 items for the global, problem-solving and support reading strategies, respectively). The participants were 155 undergraduates at Universiti Sultan Zainal Abidin in Malaysia. The findings indicated that the problem-solving strategy was used most frequently [42]. also employed a questionnaire on metacognitive reading strategies to assess the attitudes of students from Majmaah University in Saudi Arabia toward the use of online reading strategies in their development of reading comprehension. The questionnaire contained 38 items (18, 11 and 9 items for the global, problem-solving and support reading strategies, respectively). The results demonstrated no significant difference between the three online reading strategies, which was an unexpected finding.

To assess the use of online reading strategies among students, researchers use different programs for online reading texts. For example [60], employed a web-based reading strategy training program based on four types of reading strategy (i.e., global, problem-solving, support and socio-affective). A total of 72 participants (40 teachers and 32 EFL students in Taiwan) completed the assessment program. The result illustrated that only the most skillful readers make frequent use of the global reading strategy. Nevertheless, the students mostly appreciated the support reading strategy based on their attitude toward reading strategies. Furthermore [11], used the OSORS to assess the use of online reading strategies among 32 native English speakers from the northeastern United States. The questionnaire was composed of 38 items for assessing reading strategies. The findings indicated that readers tend to interpret appropriate information using context clues, background schemata and related materials; identify reading objectives; classify key information from the reading passage; balance reading speed to successfully comprehend the text; and modify necessary information to derive the correct meaning of the reading text.

In summary, these studies mainly used two types of assessment tools: (1) OSORS and (2) a developed assessment program for online reading strategies. Based on the target context, researchers assessed various reading strategies. Nevertheless, the most commonly assessed online reading approaches are the global, problem-solving and support reading strategies.

### 2.8 Students’ attitudes toward online reading

Students' attitudes toward online reading indicate whether or not they appreciate online reading [75]. Attitude toward reading is described as the mindset related to feelings that lead to an interesting or boring reading experience [76]. [63] used the [77]'s (2011) PISA – 2009 survey on digital reading literacy, which demonstrated that students' enjoyment of reading in their native language exerts a significant and positive effect on online reading achievement. Furthermore [78], conducted a study on the perspectives of Indonesian students on digital reading comprehension. Students from urban areas agreed that digital reading was extremely easy and practical to understand, whereas students from rural areas reported difficulties in online reading. As online reading uses hyperlinks and various hypermedia, the students enjoyed and actively participated in online learning [[27,79]]. In another study [75], examined the effects of students' attitudes toward online reading on language learning. The findings indicated that the majority of participants exhibited average levels of preference for digital reading and that male students displayed more positive attitudes toward online reading than female students.

In fact, these studies demonstrated that students display various attitudes toward online reading, which could influence reading comprehension achievement. Moreover, measuring students' attitudes toward online reading can provide language teachers with several benefits, such as (1) obtaining knowledge of students’ interest in reading strategies and enhancing reading comprehension, (2) assessing the effect of changes in their attitudes on language learning and (3) examining the effect of their attitudes on classroom and school experiences [80].

### 2.9 Rationale for the current study

The literature on reading strategies has predominantly focused on L_2_ reading, particularly in the online context. Numerous studies have explored the various strategies employed by L_2_ readers to enhance their comprehension when reading online [26,42,61,75]. These investigations have contributed significantly to our understanding of the role of reading strategies in L_2_ reading and have provided valuable insights into instructional practices. Despite the wealth of research in the field of L_2_ reading strategies in the literature review above, studies specifically on reading strategy use in L_1_ online reading comprehension are relatively scarce. This scarcity of research is surprising, considering the prevalence and importance of L_1_ reading in various academic and professional settings. As online platforms continue to shape the way we consume written information, it is crucial to investigate the reading strategies employed by L_1_ readers and their implications for comprehension in this context.

In summary, this literature review suggests certain ideas: (1) understanding the importance of online reading literacy encourages valuable research in online reading literacy; (2) investigating the different types of online reading strategies suggests that the three most common approaches are the global, problem-solving and support reading strategies; (3) examining methods for assessing the use of online reading strategies promotes inquiry on the use of OSORS for the present study and the influence of such strategies on reading comprehension achievement; (4) examining students' attitude toward online reading presents new ideas on whether or not their attitude toward language learning influences their use of online reading strategies, language arts achievement and reading comprehension skills; and (5) within the framework of SRL theory, numerous interconnected factors, including cognitive, metacognitive, motivational, emotional and behavioural aspects, form a comprehensive framework that impacts students’ use of online reading strategies, as well as their reading comprehension and literacy achievement. Based on this background, the present study aimed to contribute to the current research by addressing the following questions and hypotheses.

### 2.10 Research questions

RQ1 What types of online reading strategies are most frequently used by lower secondary students?

RQ2 What is the effect of students' attitude on their use of online reading strategies?

RQ3 Does students' use of online reading strategies exert any significant impact on their test results in reading comprehension and language arts achievement in L_1_?

RQ4 Is students' language arts achievement significantly related to their test results in reading comprehension?

### 2.11 Research hypotheses

RH1: Problem-solving strategies were most frequently used by lower secondary students [[12,22,74]].

RH2: Students’ grammar attitude has a positive relation with their use of online reading strategies [78].

RH3: Students’ use of online reading strategies has significant impacts on their reading comprehension achievement and language arts achievement in L_1_ [13,22].

RH4: Students’ language arts achievement is significantly related to their reading comprehension [81,82].

**Fig. 1:**
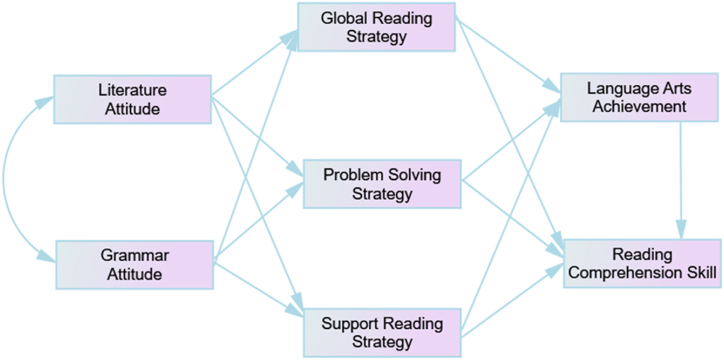
Proposed research model.

Based on these questions and hypotheses, the study proposes a research model (Fig. 1). The structural model presents four main factors, of which two are subdivided into further variables: the two exogenous variables, attitude toward L_1_ language literature and grammar, and the three approaches (the global, problem-solving and support reading strategies). The endogenous variables are language arts achievement and reading comprehension test results. The study assumes twelve direct structural effects: the direct effects of both attitudinal factors on learning strategies and those of the strategies on language arts and reading comprehension test results.

## 3 Methods

### 3.1 Participants

The sample was composed of lower secondary students (N = 4527; male = 2025 and female = 1933; missing = 569) (Table 1). Missing data were random (MAR), so we used the mean imputation technique to handle the missing data. This method ensures that we can use the available data to provide valid and unbiased estimates. The Hungarian education system includes three levels. The first is the primary level, where education lasts from Years 1–8. It has two main phases, the elementary and lower secondary levels. The present research focused on students in Years 5–8 aged 11–14 years. In the first two years, language arts mainly comprise learning the basics of reading and writing skills. By the third year, language arts are expanded to include grammar and literature. The students attend four to five language arts lessons per week.

**Table 1:** Participants in the study.

Year	Gender			Total
	Boys	Girls	Missing gender	
5	401	380	17	798
6	600	620	35	1255
7	414	398	20	832
8	609	535	16	1160
Missing year	1	0	481	482
Total	2025	1933	569	4527

### 3.2 Instruments

#### 3.2.1 School marks in language arts

Language arts achievement was indicated by the students' school marks in this subject. The language arts subject is composed of two major parts, Hungarian grammar and literature. Students’ different achievement levels were evaluated on a five-point scale based on the Hungarian evaluation system (5 = Excellent; 1 = Weakest).

#### 3.2.2 Questionnaire for attitude toward language arts

Attitude was measured using a self-response questionnaire item. The students were asked to answer the following question: To what extent do you like language arts? When evaluating language arts, the assessment encompasses two primary components, one's perspective on literature and one's approach to grammar. Responses were rated on a four-point Likert scale (1 = Strongly dislike; 4 = Strongly like).

#### 3.2.3 Reading comprehension test

Reading comprehension was measured with two main tasks. In the first part, the semantics of words were measured using cloze tests, in which words and phrases essential to the comprehension of course materials were included. In the second session, a brief passage was provided to assess reading comprehension skills likewise with cloze test items. These items gauged students' literal understanding of the reading text, in alignment with Barrett's taxonomy [83]. The complete test comprised a total of 44 items. Its reliability was deemed satisfactory with a Cronbach's alpha value of 0.87. As regards the reading equivalency level, the passages on the English-language reading test were selected to align with the B2 level in accordance with the Common European Framework of Reference for Languages (CEFR) [84]. Additionally, it is important to note that the passages were of an expository nature and contained a total of 675 words.

#### 3.2.4 Online survey of reading strategies

In order to assess online reading strategies, the study used 38 items sourced from the online survey of reading strategies (OSORS) developed by Ref. [12]. These items were employed to measure metacognitive approaches, with 18, 11 and 9 items dedicated to evaluating global, problem-solving and support reading strategies, respectively. Example items include: “I think about what I know to help me understand what I read online” (global strategies); “I try to picture or visualize information to help remember what I read online” (problem-solving strategies); and “I take notes while reading online to help me understand what I read” (support reading strategies). Responses were rated on a five-point Likert scale (1 = “I never or almost never do this when I read online”; 5 = “I always or almost always do this when I read online”). The overall rating indicates the frequency of reading strategy use, and the average for each subscale shows which approaches (i.e., the global, problem-solving or support reading strategies) are most frequently used while reading an academic text [34]. confirmed the reliability index (Cronbach's alpha) for OSORS, which reached 0.85, 0.80 and 0.75 for the global reading, problem-solving and support reading strategies, respectively.

#### 3.2.5 Procedure

The study used the quantitative research method. The OSORS questionnaire and an online reading comprehension test were administered via the eDia online testing platform (edia.hu), which is maintained by the University of Szeged Centre for Research on Learning and Instruction [85]. Students from the participating schools are familiar with this system because several online assessments have already been conducted on the platform.

Ethical approval was obtained from the Institutional Review Board at the Doctoral School (University of Szeged, No 8/2017). Students whose parent or legal guardian provided written informed consent were included in the research. Written informed consent was administered by the students’ school. Data were treated confidentially, and disclosure to third parties was prohibited. To ensure data handling, the students were assigned identification codes, and only the school administrator was able to identify the students with their results. The researchers lacked access to any personal information to maintain anonymity.

Participation of the schools and students was voluntary. Data collection covered one school lesson, which lasted approximately 40 min. During the testing procedure, the researchers presented information about the research, and then the students completed the attitude questionnaire and indicated their school marks and background data (e.g., school, year and gender). Afterward, they completed the reading comprehension test. Finally, they completed the questionnaire on online reading strategies. The students were encouraged to work independently and at their own pace. Automatic correction was provided for the test, such that the students received immediate feedback on their performance and the schools were provided with the results.

#### 3.2.6 Data analysis

The study used three methods for data analysis. The first is classical test analysis using SPSS for Microsoft Windows 2.0, which consisted of an estimation of the internal consistency of the questionnaire at the subscale level and an estimation of frequencies, means and standard deviations. Second, a Rasch analysis was employed with the help of Quest and WINSTEPS. This method was used to investigate the person–item fit of the questionnaire and the difficulty levels of the items on the reading comprehension test. Acceptable average infit mean square (INFIT MNSQ) and outfit mean square (OUTFIT MNSQ) scores ranged from .5 to 1.5. The acceptable scores for infit z-standardized (INFIT ZSTD) and outfit z-standardized (OUTFIT ZSTD) ranged from −2 to +2 [86]. The Chi-squared value indicates that the data met the normal distribution criteria (χ^2^/*df* < 3; [87]. Third, the study used confirmatory factor analysis (CFA) to confirm the structure of the related factors from the questionnaire, whereas path analysis was employed to identify the effects between constructs. Path analysis is a convenient method to test relations between hypothesized models and to identify direct and indirect relations and their directions [88]. One-headed arrows indicate direct paths, whereas double-headed arrows denote reversible relationships between them. To justify the model fit, CFA and path analysis were conducted with the SPSS AMOS 20 software package [89]. To estimate the model fit, the study computed the Tucker–Lewis index (TLI), normed fit index (NFI), comparative fit index (CFI) and root mean square error of approximation (RMSEA). These indicators ranged between 0 and 1 with large values pointing to a better fit. In the cases of TLI, NFI and CFI, a cut-off value of 0.90 or higher indicates an acceptable model fit. RMSEA, which specifies the level of difficulty of the structured model, ranges from 0 to 1 (acceptable <.80, [88]).

#### 3.2.7 Reliability and validity of instruments

We confirmed the reliability of persons and items for the OSORS questionnaire and measured its internal consistency (Cronbach's alpha). At the subscale level, these values reached 0.89, 0.86 and 0.82 for the global, problem-solving and support reading strategies, respectively.

A Rasch analysis was performed to confirm the consistency of the students' responses and the appropriateness of the items. Table 2 presents a Cronbach's alpha value (KR-20) of 0.95, which points to the level of interaction between persons and items. Furthermore, for the person measure, the scores for INFIT MNSQ and OUTFIT MNSQ were the same (1.04), which is acceptable. The score for INFIT ZSTD and OUTFIT ZSTD was −0.3. The person separation score was 3.49, which indicates that the data consist of three levels, low, medium and high performance. The Chi-squared score showed that the data met the normal distribution criteria (χ^2^/*df* < 3); thus, the Rasch model fitted globally.

**Table 2:** Measures of persons and items in OSORS.

Measures	Persons	Items
Number	4527	38
Reliability	.92	.93
Mean	117.30	10,881.00
Standard deviation	28.20	1209.10
Model error	.17	.02
Infit mean square (MNSQ)	1.04	1.00
Infit z-standardized (ZSTD)	−.3	−1.3
Outfit mean square (MNSQ)	1.04	1.04
Outfit z-standardized (ZSTD)	−.30	−.80
Separation	3.49	19.433
Overall test reliability, Cronbach's alpha (KR-20)	.95	
Chi-squared (*χ*^2^)	343,207.31	
*df*	123,166	
*p*	<.001***	

The data confirmed the reliability for persons (0.92) and items (0.93). In other words, the difficulty levels of the items were consistent with the students’ responses; the test quality was therefore sufficient. Thus, the questionnaire could be assumed to be a good assessment for fulfilling the requirements of the Rasch analysis. The item separation score (which indicates the difficulty levels of the items) was 19.43. Hence, the study concluded that the instrument was excellent for measuring the use of online reading strategies among students during reading comprehension tasks.

The study also identified the fit statistics for items rated on a four-point Likert scale to examine the students' attitudes toward L_1_ language arts. For these statistics, we used Rasch analysis (Conquest). The weighted MNSQ was analysed for the item on students' attitudes toward language arts achievement in L_1_. In this study, the weighted MNSQ of this item on attitude is 0.49 (acceptable range: 0.5–1.5, [86]); we therefore find that the expected MNSQ (dotted line) and weighted MNSQ (solid line) are only slightly different (Fig. 3). We thus deem that the attitude–investigation item is well-fitted, free from redundant information and appropriate; moreover, the responses to and appropriateness of the item are nearly consistent in the measurement of the students’ attitudes toward Hungarian language arts.

Knowledge of the reliability of the reading comprehension test is necessary to address RQ3. The reliability estimates (Cronbach's alpha) were 0.84, 0.74 and 0.78 for the entire test, the semantic part and the reading comprehension part, respectively. The study also investigated the difficulty level of the items on the reading comprehension test and the characteristics of the students' language arts achievement/test.

First, we investigated the difficulty levels of the items on the reading comprehension test. Using Rasch analysis, we constructed an item–person map (Fig. 5) to describe the item difficulty of the test and the achievement levels of the students. The left panel of the map indicates student achievement, which points to high achievement in the upper part and low achievement in the lower part. The right panel focuses on the difficulty level of the items, which highlights difficult items in the upper part and easy items in the lower part. Fig. 5 indicates that student achievement displays a normal distribution (because the high level of achievement is nearly centred at the logical point, that is, 0). Therefore, the study suggests that the items on the reading comprehension test were neither extremely difficult nor easy.

Second, we used Rasch analysis and launched the Quest program to investigate the test characteristics of the students' language arts achievement. The test uses the following grading system (1 = fail; 2 = acceptable; 3 = satisfactory; 4 = good; 5 = excellent). Fig. 6 depicts the test characteristics of the Hungarian students’ language arts achievement. The important characteristics are described using the INFIT MNSQ for the test question. Its measurement is based on item fit/consistency to the item characteristic curve by considering its weight or actual content in accordance with the accurate probability level (0.5; [90]). Its value (weighted MNSQ) for this test item is 1.39 (between the acceptable range of 0.5–1.5; [86]). Therefore, the study concludes that the test for L_1_ language arts achievement is well-fitted for the students.

## 4 Results

### 4.1 RQ1

To address RQ1, the study computed the descriptive statistics for the OSORS questionnaire (Table 3). Mean scores and standard deviations indicated a moderate use of strategies for the total number of questionnaires using various factors; the problem-solving strategy was found to be the most used in all years.

**Table 3:** Descriptive statistics for the OSORS.

Year	Global reading M (SD)	Problem-solving M (SD)	Support reading M (SD)	Total M (SD)
5	3.20 (.77)	3.43 (.82)	3.25 (.87)	3.28 (.76)
6	3.18 (.73)	3.47 (.78)	3.27 (.83)	3.29 (.72)
7	3.08 (.76)	3.34 (.81)	3.06 (.84)	3.15 (.75)
8	3.07 (.78)	3.27 (.82)	2.99 (.86)	3.11 (.77)
Total	3.14 (.76)	3.38 (.81)	3.15 (.86)	3.21 (.75)

**Fig. 2:**
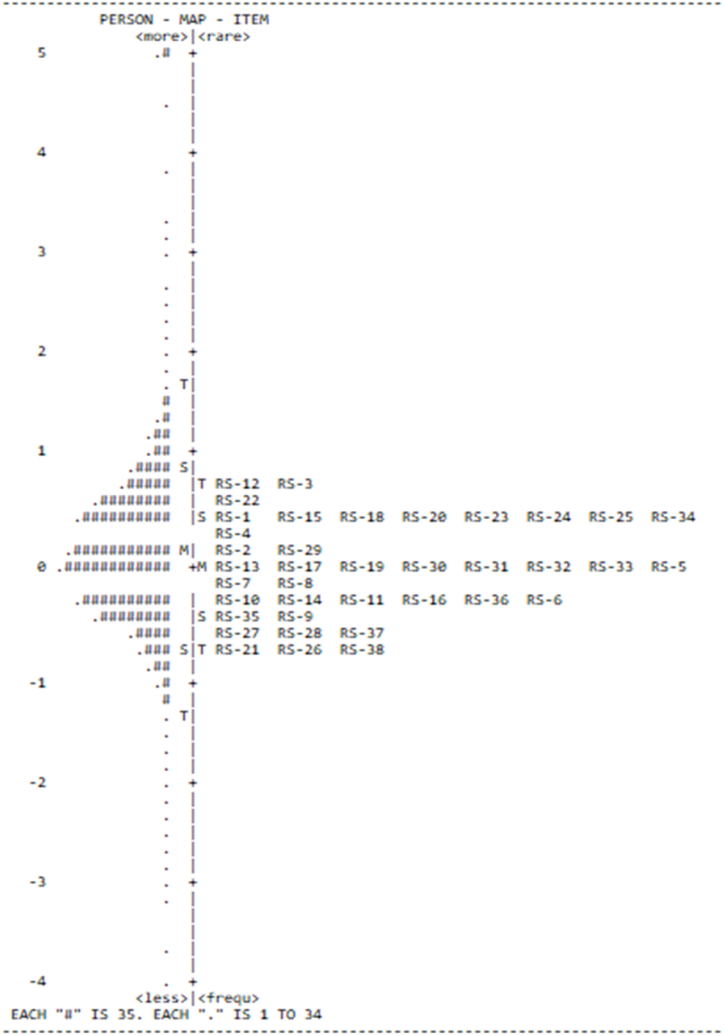
Wright map of the questionnaire items on the use of online reading strategies (N = 4527).

To specify the use of items based on the reading strategy questionnaire, we also used Rasch analysis and ran WINSTEPS (Fig. 2). The left panel of the figure indicates participants who used the items (more or less) from the online reading text, whereas the right panel describes the use of items (rarely and frequently). The upper part of the left panel indicates students who used the reading items from the survey questionnaire (more), and the lower part shows the ones who used the reading items (less). The upper part of the right panel denotes items rarely used by the students, and the lower part indicates items frequently used by the students as good online reading strategies. The findings suggested that the students used items 21 and 26 most frequently (those related to the problem-solving strategy) and used less of the global reading strategy (items 3 and 12). This result implied that they enjoyed elucidating confusing information through their interpretation based on background knowledge. The study also found that the students appreciated the support reading strategy (item 38). Furthermore, it denoted that the students are keen to highlight key information and take notes to summarize ideas in reading passages.

**Fig. 3:**
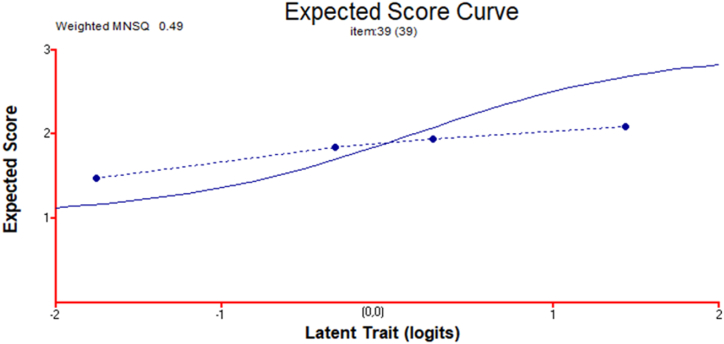
Item characteristic curve for the attitude of students toward Hungarian language arts (N = 4527).

### 4.2 RQ2

Certain variables were related to the use of reading strategies, such as attitude toward language arts (including literature and grammar), language arts achievement and reading comprehension skills. Therefore, means and standard deviations were also computed to estimate attitudes toward language arts, language arts achievement and the results for the reading comprehension test (Table 4). These values were higher for students in Years 5 and 6 and lower for those in Years 7 and 8. Language arts achievement was relatively high at 4.04, thus indicating positive marks in this subject. The results of the reading comprehension test ranged from 69.23 % to 77.14% with an average of 73.20 %.

**Table 4:** Descriptive statistics of variables.

Year	Attitude toward language arts (literature and grammar) M (SD)	Language arts achievement M (SD)	Reading comprehension test results (%p) M (SD)
5	2.95 (.66)	4.28 (.77)	69.23 (14.12)
6	2.74 (.67)	3.98 (.88)	71.40 (13.90)
7	2.67 (.66)	4.01 (.87)	75.03 (13.42)
8	2.68 (.68)	3.96 (.91)	77.14 (14.90)
Total	2.75 (.68)	4.04 (.87)	73.20 (14.40)

We conducted a path analysis to investigate the possible effect of the constructs of the hypothesized model. The results did not confirm our hypothesis (Chi-squared = 0.000, *df* = 0, *p*-value cannot be computed, CFI = 1.000, TLI = no data, NFI = 1.000, RMSEA = 0.178). Therefore, we modified the model and omitted the direct path between attitude and the results on the reading comprehension test. The decision to remove this path was based on the higher modification index identified by the MPlus software compared to other potential paths. Following the deletion, the model analysis (Table 5) demonstrated a satisfactory CFI value of 0.996. Then, TLI = 0.961 and NFI = 0.995 further confirmed that the constructed model fits the data well. RMSEA also displayed a very good value of 0.035.

**Table 5:** Fit indices of the confirmed model.

Event	*χ*^2^	*Df*	*p-*Value (>.05)*	SRMR (<.05)*	NFI (≥.9)*	CFI (≥.9)*	TLI (≥.9)*	RMSEA (<.08)*
Path analysis of the confirmed model	6.558	1	.014	.036	.995	.996	.961	.035

**Fig. 4:**
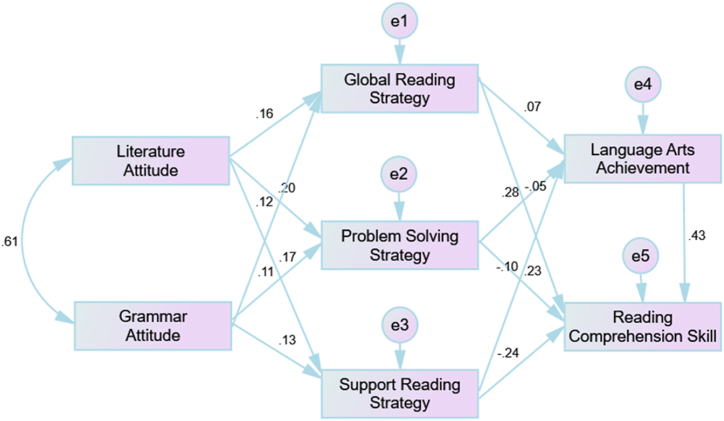
Confirmed model of the research (N = 4527).

The confirmed model (Fig. 4) depicted the indirect effect of students’ attitudes toward language arts via the use of online reading strategies. Specifically, attitude toward literature and grammar exerted positive and significant effects on the use of the three approaches, the global (*β* = 0.16 and 0.12; *p* < .05), problem-solving (*β* = 0.20 and 0.11; *p* < .05) and support (*β* = 0.17 and 0.13; *p* < .05) reading strategies. In other words, the students were likely to use more reading strategies if they had a positive attitude toward language arts learning (literature and grammar).

**Fig. 5:**
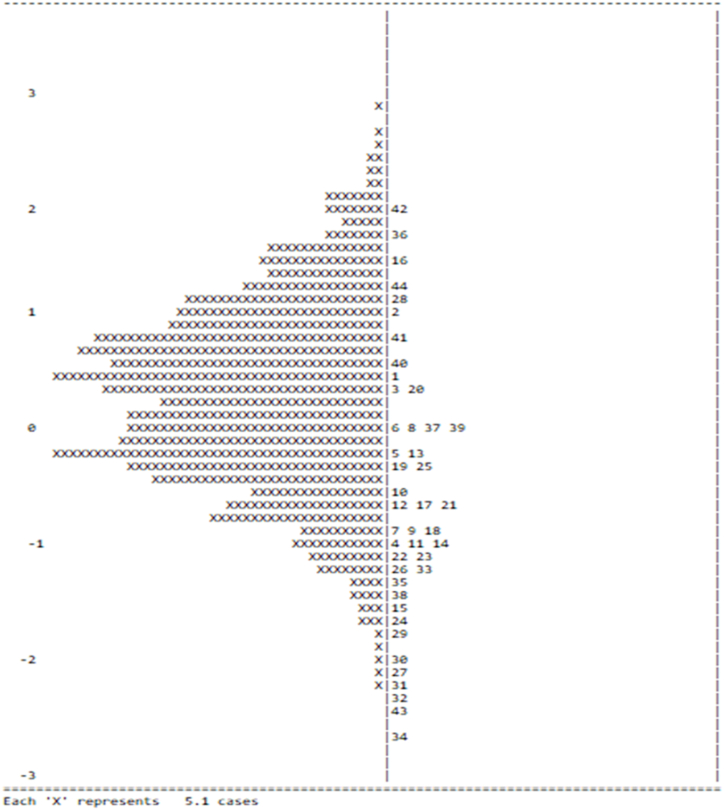
Item–person map describing the difficulty levels of the items of the reading comprehension text (N = 4527).

**Fig. 6:**
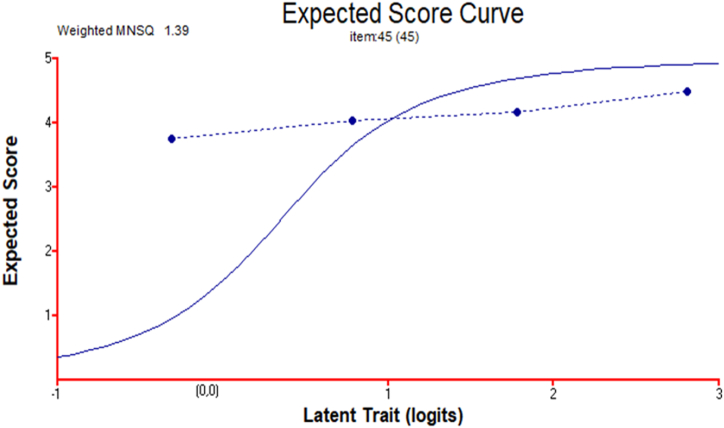
Test characteristics of the language arts performance of Hungarian students.

### 4.3 RQ3

After investigating the difficulty levels of the items for the reading comprehension test and the test characteristics of the students' language arts achievement, the study sought the answer to RQ3, which is related to the impact of the use of students’ online reading strategies on reading comprehension achievement. The confirmed model (Fig. 4) demonstrated that the use of problem-solving strategies exerted significant and positive impacts on reading comprehension achievement, explaining approximately 42 % of the variance (*β* = 0.23; *p* < .05). Nevertheless, the study found no significant effect for the two other types of reading strategies (global: *β* = −0.05; *p* > .05; support: *β* = −0.24; *p* > .05) on reading comprehension achievement. As regards the use of online reading strategies on language arts achievement, the confirmed model demonstrated that the use of the problem-solving strategy had significant impacts on language arts achievement, accounting for approximately 36 % of the variance (*β* = 0.28; *p* < .05); nevertheless, no effects were observed from the use of the global (*β* = 0.07; *p* > .05) and support (*β* = −0.10; *p* > .05) reading strategies. There may be certain reasons for these results, such as the preference of other Hungarian students for traditional reading (offline) over online reading, difficulty in using technological tools and lack of skill in adjusting to the reading text and objectives [27].

### 4.4 RQ4

The last research question confirmed the impact of language arts achievement on reading comprehension achievement. Fig. 4 indicates that the confirmed model displays the positive effect of the students’ language arts achievement on the reading comprehension test results (*β* = 0.43, *p* < .05) using path analysis.

Language arts achievement was based on the students’ achievement in Hungarian literature and grammar. Thus, the study applied Chi-squared statistics to confirm this result in detail. The analysis demonstrated a higher significant relation between grammar achievement and reading comprehension skills (*χ*^2^ = 0.76, *p* < .001), whereas a moderate and significant relation existed between literature achievement and reading comprehension skills (*χ*^2^ = 0.46, *p* < .001). Therefore, under the domain of Hungarian literature arts, grammar achievement exerted a more significant relation (*χ*^2^ = 0.76) on reading comprehension skills than literature achievement did (*χ*^2^ = 0.46). Hence, if students accumulate more knowledge in grammar for L_1_ learning, then they can do better on reading comprehension tasks.

## 5 Discussion

This study addressed four research questions and four respective hypotheses. It was found that our study findings supported all of these research hypotheses. The first research question focused on the online reading strategies frequently used by students. Initially, we confirmed the reliability of the OSORS questionnaire with different methods of analysis, such as descriptive and Rasch analyses. After confirmation, we examined the online reading strategies frequently used by Hungarian students for online reading. The findings showed that the most regularly employed approaches on reading comprehension tasks are the problem-solving and support reading strategies. This finding supports our first research hypothesis. There may be several reasons for this result: the students appreciated being able to solve reading problems at their own pace. They interpreted puzzling information, and they recalled background schemata on reading knowledge and combined such knowledge with current knowledge to understand the key information in the reading text [91]. This result (students’ most frequent use of the problem-solving strategy) is consistent with that of previous studies on the use of online reading strategies (e.g., Refs. [12,22,74]). Additionally, the result on the use of the support strategy as another most frequently used approach is in agreement with that of other studies [60,61]. The study found that the global reading strategy was the least used strategy among the Hungarian students. This result may be because this strategy enables students to decide which aspect to focus on while they read based on the objectives of reading [12,26]. Therefore, students require certain skills in monitoring, reviewing, synthesizing and inspecting the appropriateness of the reading context and interpreting information. Only readers with high levels of proficiency in reading succeeded in using the global reading strategy [60,92].

RQ2 is concerned with the impacts of the attitude of Hungarian students toward L_1_ literature and grammar on the use of online reading strategies. First, we explored the appropriateness of the attitude question item (including the four-point scale) with the expected score curve of the Rasch analysis. We also found that the attitude question was appropriate to the objectives of the study. The findings showed that the positive and significant effects of students’ attitudes toward L_1_ could be confirmed through their use of online reading strategies [78]. Thus, the study concludes that if students had positive attitudes toward L_1_ learning, then they could use further online reading strategies [93,94]. Additionally, their attitudes toward literature and grammar are closely related; in other words, if students appreciate L_1_ grammar learning, then they may also appreciate L_1_ literature learning. This finding confirms the second research hypothesis. It is also consistent with those of previous studies [27,63], which reported the importance of reading preferences or attitudes toward language learning in relation to the use of online reading strategies.

RQ3 covers the impact of the use of online reading strategies among Hungarian students on their literature arts achievement in L_1_ and reading comprehension skills. We first confirmed the appropriateness of the language arts and reading comprehension tests using Rasch analysis. The results confirmed the appropriateness of the result for further analyses. Using AMOS software (path analysis), we identified the impacts of students' use of online reading strategies on language arts achievement and reading comprehension skills. Out of the three online reading strategies, the use of the problem-solving strategy exerted a positive and significant impact on language arts achievement and reading comprehension achievement [[13,22]]. Our third research hypothesis is consistent with this finding. The reason for this result may be that the problem-solving strategy enhances students' understanding of grammar and semantics in Hungarian. The two other strategies, global and support reading strategies, exerted no significant impact on language arts achievement and reading comprehension achievement. This result may be due to the Hungarian students managing to answer the reading comprehension questions very well in their L_1_ because they were described in their native language [[28,29]]; thus, it was very easy for them to grasp key information from the reading tasks. The result was also in line with [29]'s (2022) study, thus showing the positive impacts of the problem-solving strategy on students' online reading comprehension achievement.

RQ4 focused on the relation between L_1_ language arts achievement and reading comprehension skills. Path analysis indicated that L_1_ language arts achievement exerted a significant and positive impact on reading comprehension skills. The study used two measurement factors (L_1_ literature achievement and L_1_ grammar achievement) under the concept of L_1_ language arts achievement. Therefore, Chi-squared analysis aided in identifying which factors had more positive and immediate effects on reading comprehension achievement. The findings revealed that grammar achievement had a more significant impact on reading comprehension achievement. In other words, if students obtain more grammar knowledge in L_1_ learning, then they will comprehend the reading text very well. Therefore, this result aligns with our fourth research hypothesis. This is also in line with those of previous studies [81,82], which reported that students’ grammar knowledge is crucial to their reading comprehension skills.

This study has its limitations. First, it is limited to the use of online reading strategies for L_1_ and reading comprehension skills among Hungarian students. Therefore, a comparison should be made between the students' L_1_ and L_2_ or English language reading comprehension to examine the effectiveness of the use of online reading strategies. Second, this study involved an online survey. Additionally, we recognise the potential for bias in the self-report questionnaires used in this study. Participants' responses to these questionnaires may be influenced by social desirability bias or recall bias. Third, due to the nature of just-identified models, both the CFI and TLI may yield higher values (quite close to 1.0), suggesting a perfect fit. While these high values may seem promising, they can also be misleading, as they do not necessarily imply a model that is a perfect representation of the underlying data-generating process. The absence of any model misfit is a characteristic of just-identified models, and thus CFI and TLI values may not offer sufficiently nuanced insights into the goodness of fit of the model. To address these limitations, future research could incorporate a mixed-methods approach, including semi-structured interviews, to provide a more in-depth understanding of students’ attitudes toward L_1_ learning and their use of reading strategies.

## 6 Conclusion

In conclusion, this study confirmed the importance of students’ attitudes toward language learning in relation to reading comprehension achievement (indirectly) through the use of online reading strategies; the preferred or most frequently employed online reading strategies; the effect of the use of online reading strategies on language arts achievement and reading comprehension achievement; and the close relation between language art achievement and reading comprehension achievement. In other words, the use of the problem-solving strategy for online reading is of great importance for reading comprehension achievement. Hence, this study is beneficial for language teachers and students alike.

With regard to practical implications, the results are based on the active participation of thousands of students in the online survey. Thus, data were considered reliable and valid for interpreting the related findings. Based on the findings, we determined the importance of students' attitudes toward the use of online reading strategies, language arts achievement and reading comprehension achievement. Therefore, teachers should note these results, since they can serve as a reference for stimulating students' attitudes toward language learning. Furthermore, the results suggest possible online reading strategies for use in reading online texts. When effectively employing these online reading strategies, students became active, enthusiastic, independent and creative learners in reading comprehension tasks [42]. Students use different strategies based on their reading ability; nevertheless, the study found that the students most frequently used problem-solving and support reading strategies. Therefore, language teachers should create learning opportunities for students that enable the use of these strategies. This study provides teachers with knowledge of students' attitudes toward and usage of reading strategies, which can enable teachers to formulate methods for enhancing students' reading comprehension skills. Furthermore, teachers can incorporate explicit instruction on metacognitive reading strategies within online reading activities to support students' comprehension skills [95]. Teachers should also consider the role of students' attitudes toward grammar [96] and literature [31] in online reading comprehension. Creating a positive learning environment that fosters a love for reading and stresses the importance of grammar can enhance students’ engagement and motivation, thus leading to improved online reading skills.

In terms of theoretical implications, we acknowledge the importance of students’ self-regulated learning in their metacognitive reading strategy use. Our study provides empirical evidence to support the use of metacognitive online reading strategies (global, problem-solving and support) in an online reading context. Specifically, we have demonstrated how the explicit use of online reading strategies can enhance online reading comprehension among Hungarian language learners. Therefore, our study not only provides practical implications for educators but also contributes to the theoretical understanding of self-regulated learning and metacognitive online reading strategies. Our examination of these metacognitive reading strategies provides a foundation for exploring the interactions between these strategies. Future research could investigate how these strategies complement or compete with each other in diverse populations, offering a more comprehensive understanding of their combined effects on reading comprehension. Additionally, while our study offers insights into the impact of metacognitive reading strategies in a specific timeframe, future assessments could employ longitudinal designs to examine the development of these strategies over time. In summary, our study contributes to an understanding of metacognitive reading strategies and their impact on reading comprehension within the context of our sample. The implications outlined here suggest fruitful avenues for future research that can further refine our grasp of how these strategies can be effectively assessed and used among different populations and in different settings and how they can be tailored to them.

## Data availability statement

The data that has been used is confidential.

## Funding statement

This research and the authors were supported by the Research Programme for Public Education Development, Hungarian Academy of Sciences (grant KOZOKT2021-16) and by the University of Szeged Open Access Fund (grant number: 5963). Tun Zaw Oo was also supported by the Doctoral School of Education, University of Szeged, and by the Hungarian University of Agriculture and Life Sciences, Institute of Education, MTA-MATE Early Childhood Research Group.

## CRediT authorship contribution statement

**Anita Habók:** Writing - review & editing, Writing - original draft, Visualization, Validation, Supervision, Resources, Project administration, Methodology, Investigation, Funding acquisition, Formal analysis, Data curation, Conceptualization. **Tun Zaw Oo:** Writing - review & editing, Writing - original draft, Visualization, Validation, Supervision, Resources, Project administration, Methodology, Investigation, Funding acquisition, Formal analysis, Data curation, Conceptualization. **Andrea Magyar:** Writing - review & editing, Writing - original draft, Visualization, Validation, Supervision, Resources, Project administration, Methodology, Investigation, Funding acquisition, Formal analysis, Data curation, Conceptualization.

## Declaration of competing interest

The authors declare that they have no known competing financial interests or personal relationships that could have appeared to influence the work reported in this paper.
