# Supplementary material for: The effect of reading strategy use on online reading comprehension
Source: Heliyon. 2024 Jan 12;10(2):e24281. doi: 10.1016/j.heliyon.2024.e24281 (PMC10827785; doi:10.1016/j.heliyon.2024.e24281)
Supplement: Multimedia component 1 [file mmc1.docx]

**ON-LINE SURVEY OF READING STRATEGIES**

Adapted from Kouider Mokhtari and Ravi Sheorey, 2002 by Neil J. Anderson (2003)

1. I have a purpose in mind when I read on line.

2. I participate in live chat with other learners.

3. I participate in live chat with other native speakers.

4. I take notes while reading on-line to help me understand what I read.

5. I think about what I know to help me understand what I read on-line.

6. I take an overall view of the on-line text to see what it is about before reading it.

7. When on-line text becomes difficult, I read aloud to help me understand what I read.

8. I think about whether the content of the on-line text fits my reading purpose.

9. I read slowly and carefully to make sure I understand what I am reading on-line.

10. I review the on-line text first by noting its characteristics like length and organization.

11. I try to get back on track when I lose concentration.

12. I print out a hard copy of the on-line text then underline or circle information to help me remember it.

13. I adjust my reading speed according to what I am reading on-line.

14. When reading on-line, I decide what to read closely and what to ignore.

15. I use reference materials (e.g. an on-line dictionary) to help me understand what I read on-line.

16. When on-line text becomes difficult, I pay closer attention to what I am reading.

17. I read pages on the Internet for academic purposes.

18. I use tables, figures, and pictures in the on-line text to increase my understanding.

19. I stop from time to time and think about what I am reading on-line.

20. I use context clues to help me better understand what I am reading on-line.

21. I paraphrase (restate ideas in my own words) to better understand what I read on-line.

22. I try to picture or visualize information to help remember what I read on-line.

23. I use typographical features like bold face and italics to identify key information

24. I critically analyze and evaluate the information presented in the on-line text.

25. I go back and forth in the on-line text to find relationships among ideas in it.

26. I check my understanding when I come across new information.

27. I try to guess what the content of the on-line text is about when I read.

28. When on-line text becomes difficult, I re-read it to increase my understanding.

29. I ask myself questions I like to have answered in the on-line text.

30. I check to see if my guesses about the on-line text are right or wrong.

31. When I read on-line, I guess the meaning of unknown words or phrases.

32. I scan the on-line text to get a basic idea of whether it will serve my purposes before choosing to read it.

33. I read pages on the Internet for fun.

34. I critically evaluate the on-line text before choosing to use information I read on-line.

35. I can distinguish between fact and opinion in on-line texts.

36. When reading on-line, I look for sites that cover both sides of an issue.

37. When reading on-line, I translate it into my own words.

38. When reading on-line, I think about information.
